# Synthesis, Anti-Tyrosinase Activity, and Spectroscopic Inhibition Mechanism of Cinnamic Acid–Eugenol Esters

**DOI:** 10.3390/molecules28165969

**Published:** 2023-08-09

**Authors:** Jianping Li, Xiaofeng Min, Xi Zheng, Shaohua Wang, Xuetao Xu, Jinbao Peng

**Affiliations:** 1 School of Biotechnology and Health Sciences, Wuyi University, Jiangmen 529020, Chinawyuchemxzh@126.com (X.Z.); 2 School of Pharmacy, Lanzhou University, Lanzhou 730000, China; wangshh@lzu.edu.cn

**Keywords:** tyrosinase, inhibitors, cinnamic acid, eugenol, hybrid

## Abstract

Tyrosinase plays crucial roles in mediating the production of melanin pigment; thus, its inhibitors could be useful in preventing melanin-related diseases. To find potential tyrosinase inhibitors, a series of cinnamic acid–eugenol esters (**c1**~**c29**) was synthesized and their chemical structures were confirmed by ^1^H NMR, ^13^C NMR, HRMS, and FT-IR, respectively. The biological evaluation results showed that all compounds **c1**~**c29** exhibited definite tyrosinase inhibitory activity; especially, compound **c27** was the strongest tyrosinase inhibitor (IC_50_: 3.07 ± 0.26 μM), being ~4.6-fold stronger than the positive control, kojic acid (IC_50_: 14.15 ± 0.46 μM). Inhibition kinetic studies validated compound **c27** as a reversible mixed-type inhibitor against tyrosinase. Three-dimensional fluorescence and circular dichroism (CD) spectra results indicated that compound **c27** could change the conformation and secondary structure of tyrosinase. Fluorescence-quenching results showed that compound **c27** quenched tyrosinase fluorescence in the static manner with one binding site. Molecular docking results also revealed the binding interactions between compound **c27** and tyrosinase. Therefore, cinnamic acid–eugenol esters, especially **c27**, could be used as lead compounds to find potential tyrosinase inhibitors.

## 1. Introduction

**Figure 1:**
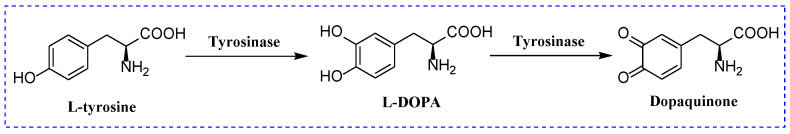
Reaction process involving tyrosinase as a catalytic protein.

Tyrosinase (EC 1.14.18.1), which is one kind of copper-containing metalloenzyme, widely exists in nature animals and plants [1,2,3]. Tyrosinase plays crucial roles in mediating the production of melanin pigment via two steps [4,5,6,7,8]. In the melanin process, tyrosinase monophenolase firstly catalyzes the hydroxylation of L-tyrosine to yield L-DOPA, which is further oxidized into *o*-dopaquinone by tyrosinase diphenolase (Figure 1) [9,10,11,12]. The produced melanin is distributed in the keratinocytes of human skin and hair; it is very important for human health [13,14,15,16,17], due to the effects of melanin in protecting human skin and eyes from damage against UV light [18,19]. However, overproduction of melanin results in epidermal pigmentation, thereby causing skin hyperpigmentation disorders, including melasma, age spots, and freckles [20,21,22]. Inhibiting tyrosinase activity can reduce the formation of melanin; thereby, tyrosinase inhibitors can be utilized to prevent melanin-related diseases [23,24]. In addition, tyrosine inhibitors also can be utilized as effective food preservatives to delay food browning [25,26,27].

**Figure 2:**
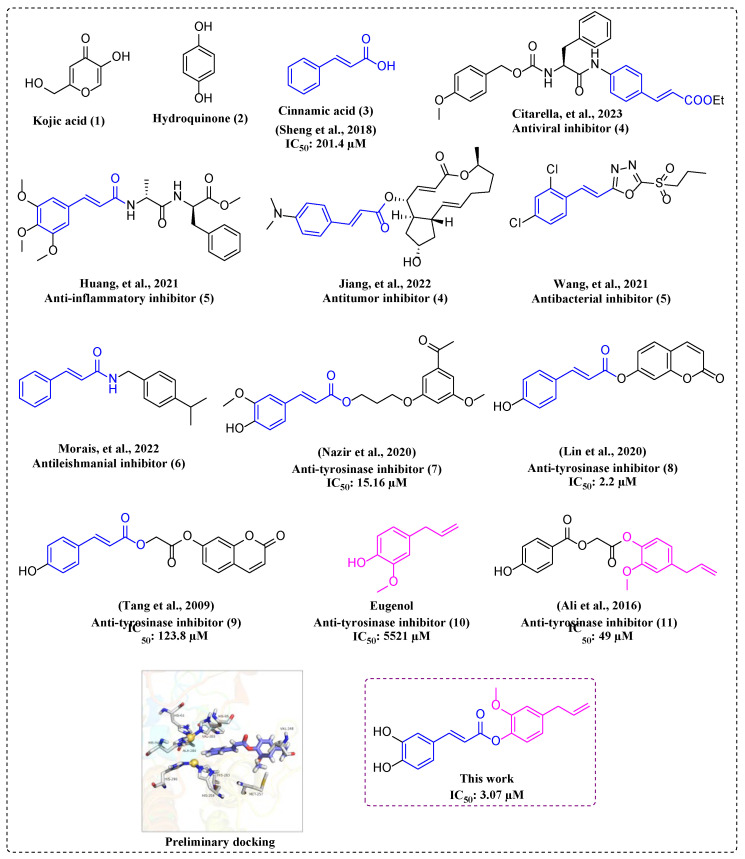
The structures of cinnamic acid, eugenol, and their derivatives, and preliminary docking [37,38,39,40,41,42,43,44,45,47].

Up to now, numerous natural and synthetic tyrosinase inhibitors have been reported [28,29,30,31,32], but only a few are used to prevent melanin production, such as kojic acid and hydroquinone (Figure 2), in spite of their instability and undesirable side effects. The abovementioned problems have prompted researchers to finding potential tyrosinase inhibitors to improve human health.

It is widely known that natural products are an important source for new drug development, and many clinical drugs are derived from natural products. Nowadays, many natural compounds have been verified to have tyrosinase inhibitory activity [33,34,35,36]. For example, cinnamic acid (Figure 2) is one important active ingredient of *Cinnamomum cassia* Presl and its derivatives promise potential tyrosinase activity [37], as well as other pharmacological activities, including antiviral [38], anti-inflammatory [39], antitumor [40], antibacterial [41], and antileishmanial activities [42] (Figure 2). Although cinnamic acid has been used in the food and medicine industries, its moderate tyrosinase inhibitory activity limits its wider application, so it is necessary to optimize cinnamic acid’s structure to enhance its tyrosinase inhibitory activity. Now, many cinnamic acid derivatives, especially cinnamic acid esters, are being designed as potential tyrosinase inhibitors [43,44,45] (Figure 2). Analyzing the above structure, it can be seen that esterification modification of cinnamic acid would be a feasible strategy to obtain better tyrosinase inhibitors, and esterification modification of the phenolic hydroxyl group would be better than a structure with an alkane chain as the bridge.

Eugenol (Figure 2), one primary bioactive component of *Eugenia caryophμllataThunb*, has shown certain tyrosinase inhibitory and antioxidant activities [46]. Cinnamic acid–eugenol esters with an alkane chain bridge have been synthesized as tyrosinase inhibitors [47] (Figure 2). Thus, we inferred that esterification modification of cinnamic acid with the phenolic hydroxyl group of eugenol would obtain more active tyrosinase inhibitors. From preliminary docking studies, a skeleton compound was bound into the active pocket with the benzene ring of cinnamic acid, close to the copper ions, and formed hydrophobic interactions with Met257, Val248, Val283, and Ala286 (Figure 2), with the aim of enhancing the binding of compound with tyrosinase. These results evidenced our idea of introducing eugenol into the parental cinnamic acid.

In addition, hybrids of pharmacophoric moieties have been an important part of the strategy to design new compound molecules with increased affinity and efficacy [24,48]. Therefore, as part of our ongoing efforts to find more potential tyrosinase inhibitors, the hybrids of the natural products cinnamic acid and eugenol were synthesized, followed by anti-tyrosinase activity screening, spectroscopic mechanism investigation, and a docking study.

## 2. Results and Discussion

### 2.1. Chemistry

**Scheme 1:**
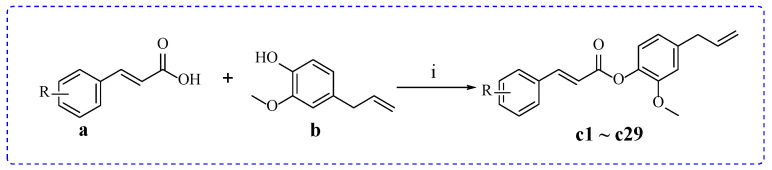
Synthesis of cinnamic acid–eugenol esters **c1**~**c29**. Reagents and conditions: (i) substituted cinnamic acids, eugenol, EDCI, DMAP, DCM, 0 °C~rt.

Cinnamic acid–eugenol esters **c1**~**c29** were conveniently synthesized using substituted cinnamic acids (a) and eugenol (b) as materials under EDCI/DMAP-mediated coupling, and the synthetic route is listed in Scheme 1. The structures of all compounds (**c1**~**c29**) were identified by FT-IR, ^1^H NMR, ^13^C NMR, and HRMS.

### 2.2. Tyrosinase Inhibitory Activity

The tyrosinase inhibitory activities of cinnamic acid–eugenol esters **c1**~**c29** were screened using L-DOPA as a substrate. We firstly screened their tyrosinase inhibition rates at a compound concentration of 64 μM. As presented in Table 1, most synthesized cinnamic acid–eugenol esters showed potential tyrosinase inhibitory activity. Among them, compounds **c14**, **c15**, and **c27** showed stronger inhibitory activities, with inhibition rates of 67.61%, 76.14%, 81.46%, while other compounds presented lower inhibition rates. Then, the 50% inhibitory concentration (IC_50_) values of compounds **c14**, **c15**, and **c27** were also investigated. As shown in Table 1, compound **c27** showed the strongest inhibitory activity, with an IC_50_ value of 3.07 ± 0.28 μM, which was stronger than that of cinnamic acid (IC_50_ = 201.4 ± 5.3 μM), eugenol (IC_50_ = 5521 ± 25 μM), and the positive control, kojic acid (IC_50_ = 14.15 ± 0.46 μM). The inhibitory activity results showed that the hybrids of cinnamic acid and eugenol, especially compound **c27**, showed stronger activities than the parent compound of cinnamic acid and eugenol, suggesting that the hybrid of cinnamic acid and eugenol might be an effective strategy to find potential tyrosinase inhibitors.

Moreover, it also was observed that the introduction of substituents group in the benzene ring of cinnamic acid resulted in a change of tyrosinase inhibitory activity. Especially, the introduction of the phenolic hydroxyl group (**c14**, **c15**, **c26**, and **c27**) obviously increased tyrosinase inhibitory activity. In particular, compound **c27** with the 3,4-diphenol hydroxy group was the most active compound (IC_50_: 3.07 ± 0.28 μM), being ~4.6-fold stronger than kojic acid (IC_50_: 14.15 ± 0.46 μM). These results showed that the introduction of the phenolic hydroxyl group might be essential for tyrosinase inhibitory activity, which is consistent with previous research results [36,37,38]. However, the introduction of non-hydroxy substituents, including methyl, chlorine, fluorine, bromine, nitro, methoxy, and trifluoromethyl, had no obvious effect in improving this activity.

### 2.3. Kinetic Study

**Figure 3:**
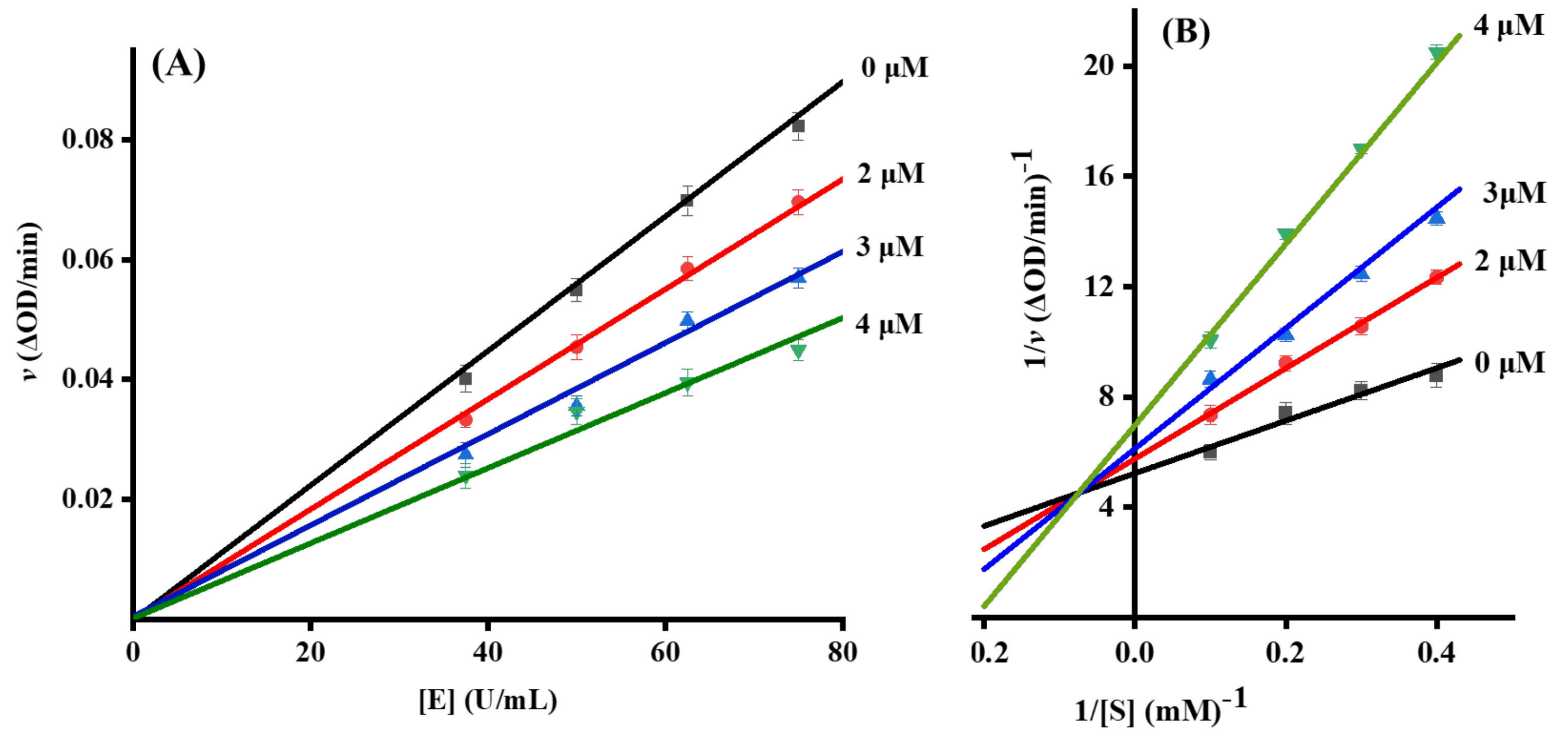
(**A**) The plots of ν versus [E] under compound **c27**; (**B**) Lineweaver–Burk plots of ν versus [S] under compound **c27**.

Inhibition kinetic studies were assayed to analyze the potential inhibition mechanism of compound **c27** toward tyrosinase. Figure 3A displays the relationship between the tyrosinase concentration with residual enzyme activity under compound **c27** (0~4 μM), which shows straight lines passing through the origin with different slopes. Therefore, compound **c27** was identified as a reversible inhibitor [49,50]. Figure 3B shows the Lineweaver–Burk plots of substrate concentration and residual enzyme activity under compound **c27** (0~4 μM). It could be observed that all lines with different slopes intersected in the second quadrant, suggesting compound **c27** is a mixed-type inhibitor [51,52].

### 2.4. Three-Dimensional Fluorescence Spectra

**Figure 4:**
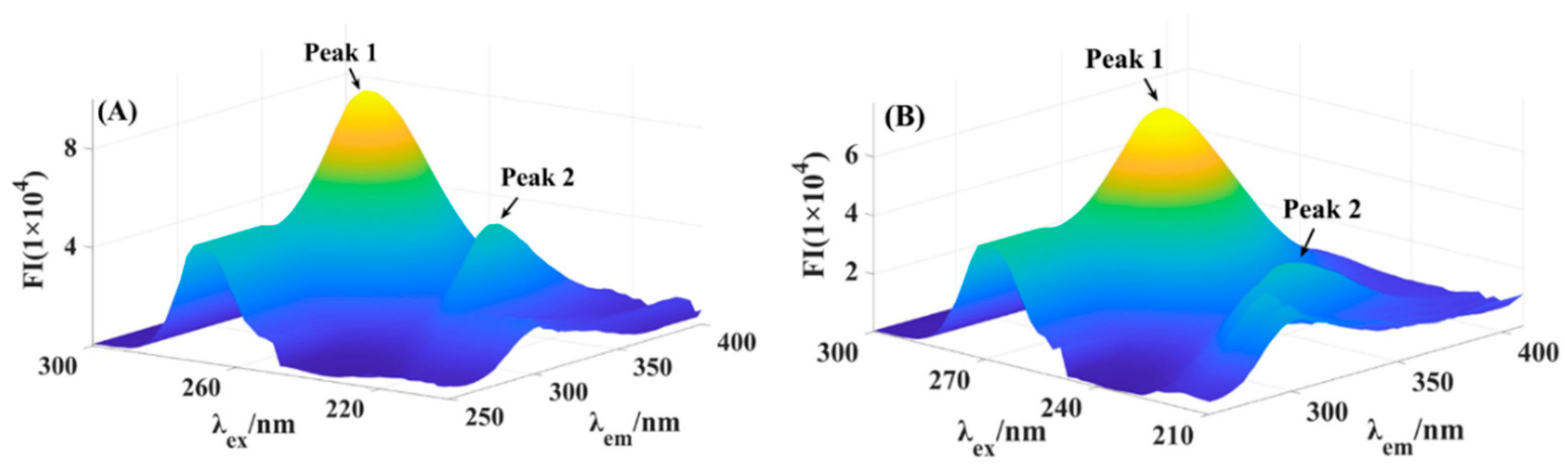
(**A**) Three-dimensional fluorescence spectra of tyrosinase. (**B**) Three-dimensional fluorescence spectra of tyrosinase with compound **c27**.

To better understand the inhibition mechanisms, the conformational change of tyrosinase by compound **c27** was monitored using a 3D fluorescence spectrum. As shown in Figure 4A, two characteristic peaks appeared, peak **1** (λ_ex_/λ_em_ = 280 nm/340 nm) and peak **2** (λ_ex_/λ_em_ = 230 nm/335 nm), corresponding to Tyr and Trp residues, and a polypeptide strand transition, respectively. Figure 4B shows the 3D fluorescence spectrum of the tyrosinase–compound **c27** mixture. It could be observed that treatment of compound **c27** reduced the intensity of peak **1** and peak **2** by 21.47% and 32.71%, respectively. These results suggest that compound **c27** could change the conformation of tyrosinase to inhibit its activity.

### 2.5. Circular Dichroism Spectra

**Figure 5:**
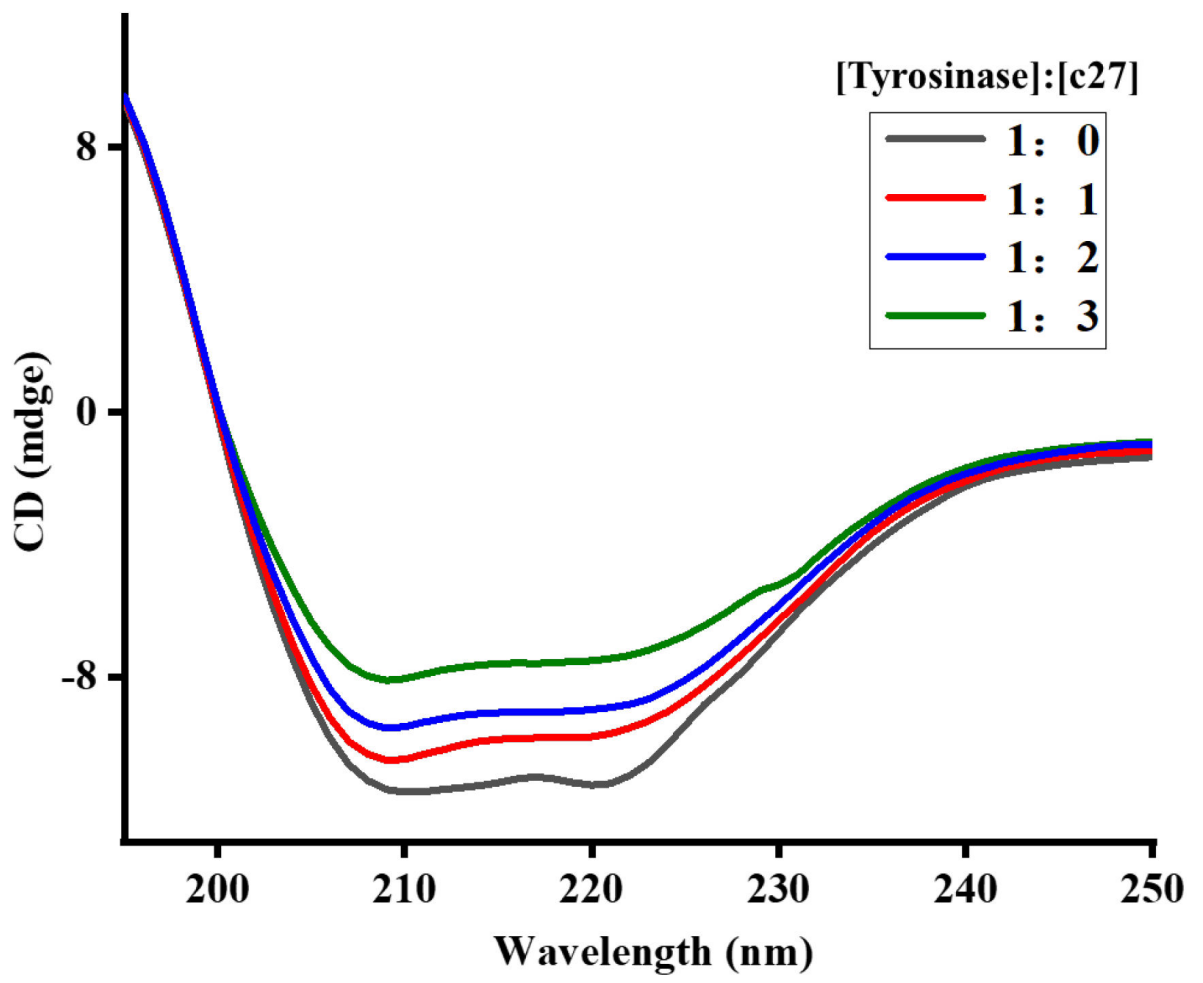
Circular dichroism spectra of the tyrosinase and compound **c27** system.

The CD spectra were also used to analyze the effects of compound **c27** on the secondary structure of tyrosinase. Figure 5 presents the CD spectra of tyrosinase–compound **c27** mixtures, which showed characteristic negative bands at 205~230 nm owing to the electronic transition of the α-helix peptide. Treatment of compound **c27** resulted in the reduction of band intensity, indicating the conformation change of tyrosinase by compound **c27**. The secondary structure contents of tyrosinase are listed in Table 2, which evidences that treatment of compound **c27** (molar ratios: 3:1) caused the increase of the α-helix (from 7.10% to 9.85%), β-sheet (from 48.90% to 49.27%), and β-turn (from 17.21% to 18.55%), but the decrease of the random coil (from 28.40% to 27.94%).

### 2.6. Fluorescence Quenching

**Figure 6:**
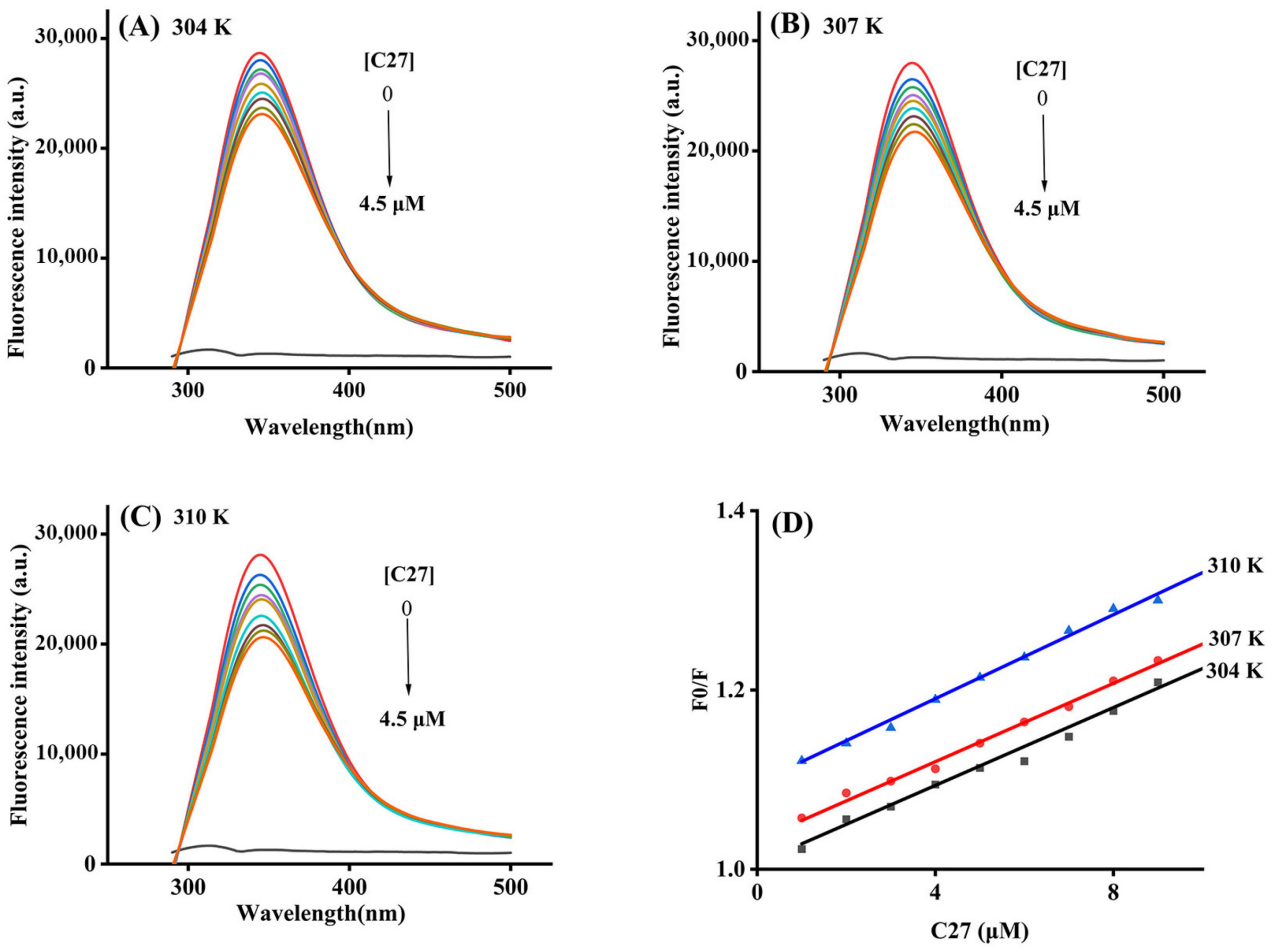
Fluorescence quenching of tyrosinase by compound **c27**. (**A**) 304 K; (**B**) 307 K; (**C**) 310 K; (**D**) Stern–Volmer plots.

Fluorescence quenching was performed to analyze the interactions between compound **c27** and tyrosinase. As shown in Figure 6A–C, the fluorescence spectra of tyrosinase at 304, 307, and 310 K reduced continuously with the titration addition of compound **c27** (0~4.5 μM), but the peak position did not shift. This phenomenon shows that the interaction of compound **c27** with tyrosinase could quench tyrosinase intrinsic fluorescence. The Stern–Volmer plots (Figure 6D) at 304, 307, and 310 K obtained from fluorescence quenching data showed good linearity, demonstrating a onefold quenching type.

To clarify the quenching mechanism, the relevant quenching constants were determined (Table 3). The Stern–Volmer quenching constant (*K*_SV_) values presented declined with the increasing temperature, and the quenching rate constant (*K*_q_) values all reached the order of magnitude of 10^12^, suggesting a static quenching process. Furthermore, the binding constant (*K*_a_) values (order of magnitude of 10^4^) suggested a moderate binding between compound **c27** and tyrosinase and the number of binding sites (n) was revealed as only one.

### 2.7. Molecular Docking

**Figure 7:**
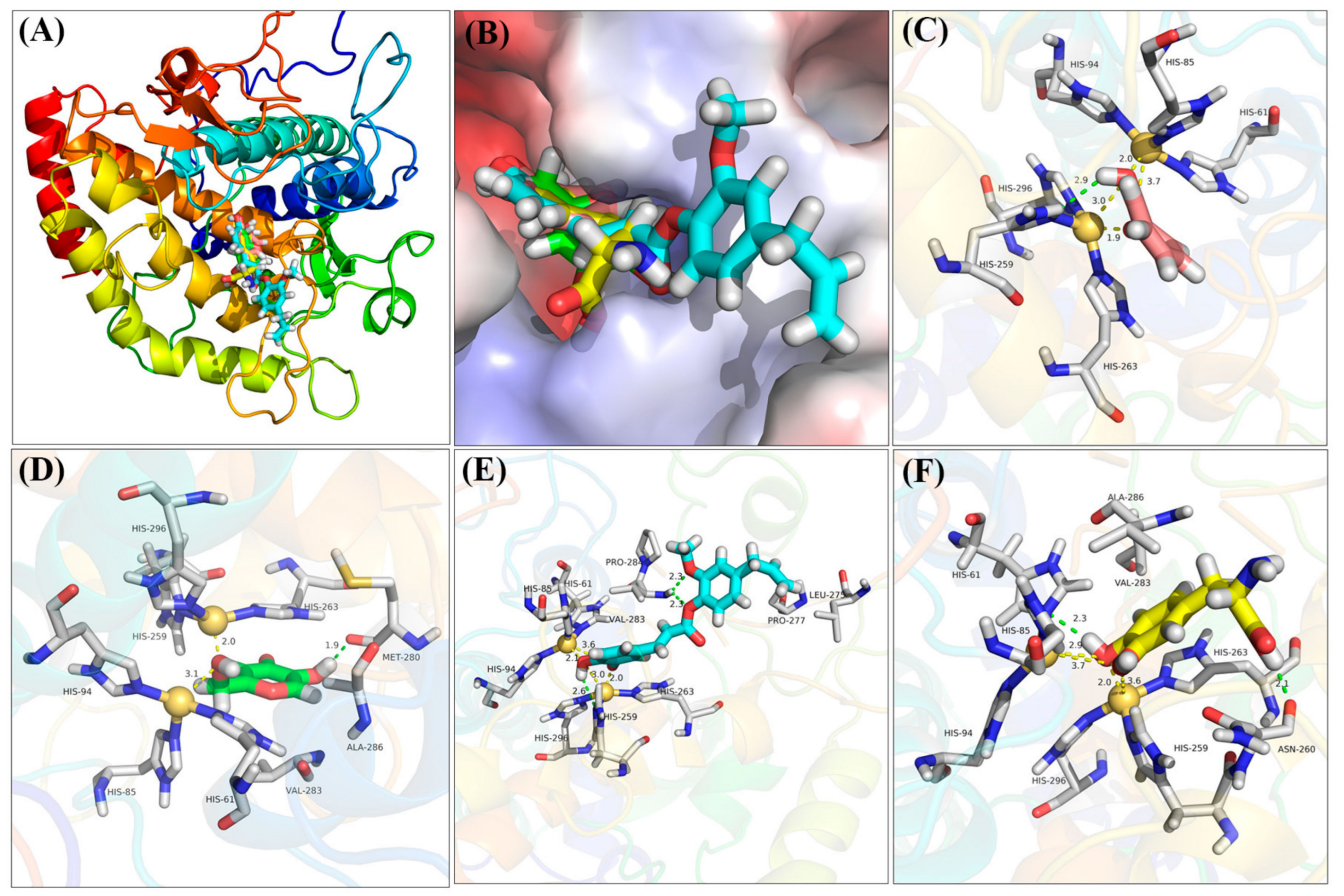
(**A**,**B**) The binding conformation of compound **c27**, tropolone, kojic acid, and L-DOPA in active pocket. (**C**) Detailed binding of tropolone with tyrosinase. (**D**) Detailed binding of kojic acid with tyrosinase. (**E**) Detailed binding of compound **c27** with tyrosinase. (**F**) Detailed binding of L-DOPA with tyrosinase.

Molecular docking was introduced to analyze the binding interaction between compound **c27** and tyrosinase, using SYBYL software. To validate the docking protocol and better understand the binding interaction of compound **c27**, analyses of the dockings of the tyrosinase (2Y9X) ligand tropolone, control kojic acid, and substrate L-DOPA with tyrosinase were also conducted, and the results are presented in Figure 6. As shown in Figure 7A,B, compound **c27**, tropolone, kojic acid, and L-DOPA were all anchored at about the same position of the active site; especially, the hydroxyl group of each compound was close to two copper ions (2.0~3.7 Å). Tropolone formed one hydrogen bond with His259 (2.9 Å) (Figure 7C), while kojic acid formed one hydrogen bond with His280 (1.9 Å) and hydrophobic interactions with His263, Val283, and Ala286 (Figure 7D). Analysis of compound **c27** (Figure 7E) showed its one phenolic hydroxyl group formed a hydrogen bond with His259 (2.6 Å), methoxy and ester groups both formed one hydrogen bond with Val283 (2.3 Å and 2.3 Å), and compound **c27** had a hydrophobic interaction with Leu275, Pro277, and Pro284, respectively. In the docking of L-DOPA (Figure 7F), there were two hydrogen bonds with His61 (2.3 Å) and Asn260 (2.1 Å), and hydrophobic interaction with His263, Val283, and Ala286. For compound **c27** and L-DOPA, the catechol rings of cinnamic acid fraction and L-DOPA were both inserted inside the cavity and located at about the same distance from two copper ions (2.0, 2.1, 3.0, 3.6 Å vs. 2.0, 2.9, 3.6, 3.7 Å). In contrast to the cinnamic acid fraction, the eugenol fraction of compound **c27** was out of the cavity. Analysis of the above interactions helps to clarify the interactions between compound **c27** and tyrosinase.

### 2.8. Drug-like Properties

The drug-like properties of compound **c27** were analyzed using SwissADME online software (https://www.swissadme.ch/index.php, accessed on 1 July 2023). As shown in Table 4, compound **c27** had favorable drug-like properties with good MW, RB, HBA, HBD, and TPSA values, except poor water solubility.

## 3. Materials and Methods

### 3.1. Materials

Tyrosinase mushroom and L-DOPA were supplied by Sigma-Aldrich (St. Louis, MI, USA). All other reagents were commercially available. ^1^H NMR and ^13^C NMR spectra were recorded on a Bruker 500 MHz NMR spectrometer instrument (Billerica, MA, USA). High-resolution mass spectral analysis (HRMS) data were measured on the Apex II by means of the ESI technique.

### 3.2. Methods

#### 3.2.1. Synthesis of Cinnamic Acid–Eugenol Ester Derivatives (**c1**~**c29**)

Substituted cinnamic acid (1.4 mmol), eugenol (1 mmol), EDCI (2 mmol), and DMAP (1.2 mmol) were added into anhydrous DCM (10 mL) under an ice bath and stirred for 30 min. Then, the mixture was stirred at room temperature until the reaction completed. Afterwards, it was quenched with water (30 mL) and the mixture was extracted with DCM (15 mL × 3). The merged organic layers were washed with saturated sodium chloride (30 mL × 3), followed by drying with anhydrous sodium sulfate, evaporating solvent, and separating by silica gel column to produce the targeted cinnamic acid–eugenol ester derivatives (**c1**~**c29**).

***4-allyl-2-methoxyphenyl cinnamate* (c1)**. White soild; yield 68% (the crude was purified using a mixture of PE/EtOAc 100:1); mp: 254.2–255.9 °C; ^1^H NMR (500 MHz, CDCl_3_) *δ* 7.88 (d, *J* = 16.0 Hz, 1H, Ar-H), 7.60 (d, *J* = 3.9 Hz, 1H, Ar-H), 7.59 (d, *J* = 2.6 Hz, 1H, CH), 7.43 (s, 1H, Ar-H), 7.42 (s, 2H, Ar-H), 7.04 (d, *J* = 8.0 Hz, 1H, Ar-H), 6.83 (s, *J* = 1.9 Hz, 1H, Ar-H), 6.81 (d, *J* = 8.0 Hz, 1H, Ar-H), 6.69 (d, *J* = 16.0 Hz, 1H, CH), 5.99 (td, *J* = 16.9, 6.7 Hz, 1H, CH), 5.17–5.08 (m, 2H, CH_2_), 3.84 (s, 3H, OCH_3_), 3.41 (d, *J* = 6.8 Hz, 2H, CH_2_); ^13^C NMR (126 MHz, CDCl_3_) *δ* 165.3 (C=O), 151.1 (Ar-C), 146.6 (CH), 139.1 (Ar-C), 138.1 (CH), 137.2 (Ar-C), 134.4 (Ar-C), 130.7 (Ar-2C), 129.1 (Ar-2C), 128.4 (Ar-C), 122.8 (Ar-C), 120.8 (Ar-C), 117.2 (CH), 116.3 (CH), 112.8 (Ar-C), 56.0 (OCH_3_), 40.3 (CH_2_); HRMS (ESI-MS) m/z: [M + H]^+^ calcd for C_19_H_18_O_3_^+^: 295.1337; found: 295.1326; IR (cm^−1^): 2937 (OCH_3_), 1704 (C=O), 1165 (Ar-C=C).

***4-allyl-2-methoxyphenyl (E)-3-(o-tolyl)acrylate* (c2)**. White soild; yield 55% (the crude was purified using a mixture of PE/EtOAc 100:1); mp: 265.1–266.3 °C; ^1^H NMR (500 MHz, CDCl_3_) *δ* 8.16 (d, *J* = 15.9 Hz, 1H, Ar-H), 7.65 (d, *J* = 7.6 Hz, 1H, Ar-H), 7.31 (t, *J* = 7.4 Hz, 1H, CH), 7.25 (s, 1H, Ar-H), 7.23 (d, *J* = 7.2 Hz, 1H, Ar-H), 7.04 (d, *J* = 7.9 Hz, 1H, Ar-H), 6.83 (s, 1H, Ar-H), 6.81 (d, *J* = 8.7 Hz, 1H, Ar-H), 6.61 (d, *J* = 15.9 Hz, 1H, CH), 5.99 (td, *J* = 16.8, 6.7 Hz, 1H, CH), 5.16–5.09 (m, 2H, CH_2_), 3.84 (s, 3H, OCH_3_), 3.41 (d, J = 6.7 Hz, 2H, CH_2_), 2.48 (s, 3H, CH_3_); ^13^C NMR (126 MHz, CDCl_3_) *δ* 165.3 (C=O), 151.1 (Ar-C), 144.2 (CH), 139.1 (Ar-C), 138.1 (CH), 138.0 (Ar-C), 137.2 (Ar-C), 133.3 (Ar-C), 131.0 (Ar-C), 130. 5 (Ar-C), 126.7 (Ar-C), 126.5 (Ar-C), 122.8 (Ar-C), 120.8 (Ar-C), 118.1 (CH), 116.3 (CH), 112.9 (Ar-C), 56.00 (OCH_3_), 40.2 (CH_2_), 20.0 (CH_3_); HRMS (ESI-MS) m/z: [M + H]^+^ calcd for C_20_H_20_O_3_^+^: 309.1487; found: 309.1483; IR (cm^−1^): 2937 (OCH_3_), 2967 (CH_3_), 1704 (C=O), 1165 (Ar-C=C).

***4-allyl-2-methoxyphenyl (E)-3-(m-tolyl)acrylate* (c3)**. Yellow soild; yield 67% (the crude was purified using a mixture of PE/EtOAc 100:1); mp: 326.6–327.8 ^°C^; ^1^H NMR (500 MHz, CDCl_3_) *δ* 7.88 (d, *J* = 16.0 Hz, 1H, Ar-H), 7.44 (s, 1H, Ar-H), 7.42 (s, CH), 7.34 (t, *J* = 7.8 Hz, 1H, Ar-H), 7.28 (d, *J* = 9.9 Hz, 1H, Ar-H), 7.07 (d, *J* = 8.0 Hz, 1H, Ar-H), 6.86 (s, *J* = 1.9 Hz, 1H, Ar-H), 6.84 (d, *J* = 8.0 Hz, 1H, Ar-H), 6.70 (d, *J* = 16.0 Hz, 1H, CH), 6.02 (td, *J* = 16.8, 6.7 Hz, 1H, CH), 5.22–5.11 (m, 2H, CH_2_), 3.87 (s, 3H, OCH_3_), 3.44 (d, *J* = 6.7 Hz, 2H, CH_2_), 2.43 (s, 3H, CH_3_); ^13^C NMR (126 MHz, CDCl_3_) *δ* 165.4 (C=O), 151.1 (Ar-C), 146.8 (CH), 139.0 (Ar-C), 138.8 (CH), 138.1 (Ar-C), 137.2 (Ar-C), 134.4 (Ar-C), 131.6 (Ar-C), 129.1 (Ar-C), 129.0 (Ar-C), 125.6 (Ar-C), 122.8 (Ar-C), 120.8 (Ar-C), 117.0 (CH), 116.3 (CH), 112.9 (Ar-C), 56.0 (OCH_3_), 40.3 (CH_2_), 21.5 (CH_3_); HRMS (ESI-MS) m/z: [M + H]^+^ calcd for C_20_H_20_O_3_^+^: 309.1487; found: 309.1484; IR (cm^−1^): 2937 (OCH_3_), 2961 (CH_3_), 1704 (C=O), 1165 (Ar-C=C).

***4-allyl-2-methoxyphenyl (E)-3-(p-tolyl)acrylate* (c4)**. Yellow soild; yield 57% (the crude was purified using a mixture of PE/EtOAc 100:1); mp: 266.8–267.7 °C; ^1^H NMR (500 MHz, CDCl_3_) *δ* 7.85 (d, *J* = 16.0 Hz, 1H, Ar-H), 7.49 (s, 1H, Ar-H), 7.48 (s, 1H, CH), 7.23 (s, 1H, Ar-H), 7.21 (s, 1H, Ar-H), 7.02 (d, *J* = 7.9 Hz, Ar-H), 6.82 (s, *J* = 1.9 Hz, 1H, Ar-H), 6.80 (dd, *J* = 8.1, 1.9 Hz, 1H, Ar-H), 6.63 (d, *J* = 16.0 Hz, 1H, CH), 5.98 (ddt, *J* = 16.8, 10.0, 6.7 Hz, 1H, CH), 5.15–5.08 (m, 2H, CH_2_), 3.83 (s, 3H, OCH_3_), 3.40 (d, *J* = 6.7 Hz, 2H, CH_2_), 2.39 (s, 3H, CH_3_); ^13^C NMR (126 MHz, CDCl_3_) *δ* 165.5 (C=O), 151.2 (Ar-C), 146.6 (Ar-C), 141.2 (Ar-C), 139.0 (CH), 138.1 (Ar-C), 137.2 (Ar-C), 131.7 (Ar-C), 129.8 (Ar-C), 128.4 (Ar-C), 122.8 (Ar-C), 120.8 (Ar-C), 116.3 (CH), 116.1 (CH), 112.9 (Ar-C), 56.0 (OCH_3_), 40.3 (CH_2_), 21.7 (CH_3_); HRMS (ESI-MS) m/z: [M + H]^+^ calcd for C_20_H_20_O_3_^+^: 309.1487; found: 309.1483; IR (cm^−1^): 2937 (OCH_3_), 2968 (CH_3_), 1704 (C=O), 1165 (Ar-C=C).

***4-allyl-2-methoxyphenyl (E)-3-(2-chlorophenyl)acrylate* (c5)**. White soild; yield 65% (the crude was purified using a mixture of PE/EtOAc 60:1); mp: 247.4–247.7 °C; ^1^H NMR (500 MHz, CDCl_3_) *δ* 8.29 (d, *J* = 16.0 Hz, 1H, Ar-H), 7.71 (dd, *J* = 7.4, 2.1 Hz, 1H, Ar-H), 7.44 (d, *J* = 7.6 Hz, 1H, CH), 7.34 (d, *J* = 7.5 Hz, 1H, Ar-H), 7.31 (d, *J* = 7.4 Hz, 1H, Ar-H), 7.04 (d, *J* = 7.9 Hz, 1H, Ar-H), 6.83 (s, *J* = 1.9 Hz, 1H, Ar-H), 6.81 (d, *J* = 8.0 Hz, 1H, Ar-H), 6.67 (d, *J* = 16.0 Hz, 1H, CH), 5.98 (ddt, *J* = 16.8, 10.0, 6.7 Hz, 1H, CH), 5.16–5.09 (m, 2H, CH_2_), 3.84 (s, 3H, OCH_3_), 3.41 (d, *J* = 6.7 Hz, 2H, CH_2_); ^13^C NMR (126 MHz, CDCl_3_) *δ* 164.8 (C=O), 151.1 (Ar-C), 142.2 (CH), 139.2 (Ar-C), 138.0 (CH), 137.2 (Ar-C), 135.3 (Ar-C), 132.6 (Ar-C), 131.45 (Ar-C), 130.4 (Ar-C), 128.0 (Ar-C), 127.3 (Ar-C), 122.7 (Ar-C), 120.9 (Ar-C), 119.8 (CH), 116.3 (CH), 112.9 (Ar-C), 56.0 (OCH_3_), 40.2 (CH_2_); HRMS (ESI-MS) m/z: [M + H]^+^ calcd for C_19_H_17_ClO_3_^+^: 329.0947; found: 329.0937; IR (cm^−1^): 2937 (OCH_3_), 1704 (C=O), 1165 (Ar-C=C), 726 (Cl).

***4-allyl-2-methoxyphenyl (E)-3-(3-chlorophenyl)acrylate* (c6)**. Yellow soild; yield 73% (the crude was purified using a mixture of PE/EtOAc 60:1); mp: 278.3–278.9 °C; ^1^H NMR (500 MHz, CDCl_3_) *δ* 7.80 (d, *J* = 16.0 Hz, 1H, Ar-H), 7.57 (s, 1H, Ar-H), 7.45 (d, *J* = 7.4 Hz, 1H, CH), 7.39 (d, *J* = 8.2 Hz, 1H, Ar-H), 7.36 (d, *J* = 7.5 Hz, 1H, Ar-H), 7.03 (d, *J* = 7.9 Hz, 1H, Ar-H), 6.82 (s, 1H, Ar-H), 6.81 (d, *J* = 8.0 Hz, 1H, Ar-H), 6.67 (d, *J* = 16.1 Hz, 1H, CH), 5.98 (td, *J* = 16.8, 6.7 Hz, 1H, CH), 5.15–5.09 (m, 2H, CH2), 3.83 (s, 3H, OCH3), 3.40 (d, *J* = 6.7 Hz, 2H, CH2); ^13^C NMR (126 MHz, CDCl_3_) *δ* 164.9 (C=O), 151.0 (Ar-C), 144.9 (Ar-C), 139.2 (CH), 138.0 (Ar-C), 137.2 (Ar-C), 136.2 (CH), 135.0 (Ar-C), 130.5 (Ar-C), 130.3 (Ar-C), 128.2 (Ar-C), 126.5 (Ar-C), 122.7 (Ar-C), 120.9 (Ar-C), 118.7 (CH), 116.3 (CH), 112.9 (Ar-C), 56.0 (OCH_3_), 40.2 (CH_2_); HRMS (ESI-MS) m/z: [M + H]^+^ calcd for C_19_H_17_ClO_3_^+^: 329.0947; found: 329.0937; IR (cm^−1^): 2937 (OCH_3_), 1704 (C=O), 1165 (Ar-C=C), 741 (Cl).

***4-allyl-2-methoxyphenyl (E)-3-(4-chlorophenyl)acrylate* (c7)**. White soild; yield 62% (the crude was purified using a mixture of PE/EtOAc 100:1); mp: 282.3–283.3 °C; ^1^H NMR (500 MHz, CDCl_3_) *δ* 7.82 (d, J = 16.0 Hz, 1H, Ar-H), 7.52 (s, 1H, Ar-H), 7.50 (d, 1H, CH), 7.40 (s, 1H, Ar-H), 7.38 (s, 1H, Ar-H), 7.02 (d, *J* = 8.0 Hz, 1H, Ar-H), 6.82 (s, 1H, Ar-H), 6.80 (d, *J* = 8.0 Hz, 1H, Ar-H), 6.64 (d, *J* = 15.9 Hz, 1H, CH), 5.98 (td, *J* = 16.9, 6.7 Hz, 1H, CH), 5.17–5.09 (m, 2H, CH_2_), 3.83 (s, 3H, OCH_3_), 3.40 (d, *J* = 6.7 Hz, 2H, CH_2_); ^13^C NMR (126 MHz, CDCl_3_) *δ* 165.0 (C=O), 151.0 (Ar-C), 145.1 (CH), 139.2 (Ar-C), 137.2 (CH), 136.7 (Ar-C), 136.6 (Ar-C), 132.9 (Ar-C), 129.6 (Ar-2C), 129.4 (Ar-2C), 122.7 (Ar-C), 120.8 (Ar-C), 117.8 (CH), 116.3 (CH), 112.9 (Ar-C), 56.0 (OCH_3_), 40.2 (CH_2_); HRMS (ESI-MS) m/z: [M + H]^+^ calcd for C_19_H_17_ClO_3_^+^: 329.0947; found: 329.0936; IR (cm^−1^): 2937 (OCH_3_), 1704 (C=O), 1165 (Ar-C=C), 755 (Cl).

***4-allyl-2-methoxyphenyl (E)-3-(2-fluorophenyl)acrylate* (c8)**. Yellow soild; yield 69% (the crude was purified using a mixture of PE/EtOAc 100:1); mp: 270.6–271.2 °C; ^1^H NMR (500 MHz, CDCl_3_) *δ* 8.00 (d, *J* = 16.2 Hz, 1H, Ar-H), 7.61 (t, *J* = 7.6 Hz, 1H, Ar-H), 7.39 (d, *J* = 8.2 Hz, 1H, CH), 7.20 (t, *J* = 7.5 Hz, 1H, Ar-H), 7.13 (d, *J* = 18.9 Hz, 1H, Ar-H), 7.04 (d, *J* = 8.0 Hz, 1H, Ar-H), 6.83 (s, 1H, Ar-H), 6.81 (d, *J* = 7.8 Hz, 1H, Ar-H), 6.77 (s, 1H, CH), 6.04–5.94 (m, 1H, CH), 5.16–5.08 (m, 2H, CH_2_), 3.84 (s, 3H, OCH_3_), 3.41 (d, *J* = 6.7 Hz, 2H, CH_2_); ^13^C NMR (126 MHz, CDCl_3_) *δ* 165.1 (C=O), 162.6 (Ar-C), 160.6 (Ar-C), 151.1 (CH), 139.1 (Ar-C), 138.1 (CH), 137.2 (Ar-C), 132.2 (Ar-C), 132.1 (Ar-C), 129.4 (Ar-C), 124.7 (Ar-C), 124.7 (Ar-C), 122.7 (Ar-C), 120.8 (CH), 119.8 (CH), 116.3 (Ar-C), 112.9 (Ar-C), 56.0 (OCH_3_), 40.2 (CH_2_); HRMS (ESI-MS) m/z: [M + H]^+^ calcd for C_19_H_17_FO_3_^+^: 313.1243; found: 313.1231; IR (cm^−1^): 2937 (-CH_3_O), 1704 (C=O), 1165 (Ar-C=C), 1317 (F).

***4-allyl-2-methoxyphenyl (E)-3-(3-fluorophenyl)acrylate* (c9).** Yellow soild; yield 45% (the crude was purified using a mixture of PE/EtOAc 100:1); mp: 279.6–280.4 °C; ^1^H NMR (500 MHz, CDCl3) δ 7.82 (d, *J* = 16.0 Hz, 1H, Ar-H), 7.39 (d, *J* = 5.5 Hz, 1H, Ar-H), 7.35 (d, *J* = 7.8 Hz, 1H, CH), 7.28 (d, *J* = 9.5 Hz, 1H, Ar-H), 7.12 (d, *J* = 9.3 Hz, 1H, Ar-H), 7.03 (d, *J* = 8.0 Hz, 1H, Ar-H), 6.83 (s, *J* = 1.9 Hz, 1H, Ar-H), 6.81 (d, *J* = 7.9 Hz, 1H, Ar-H), 6.66 (d, *J* = 16.0 Hz, 1H, CH), 5.98 (td, *J* = 16.8, 6.7 Hz, 1H, CH), 5.16–5.09 (m, 2H, CH_2_), 3.83 (s, 3H, OCH_3_), 3.40 (d, *J* = 6.8 Hz, 2H, CH_2_); ^13^C NMR (126 MHz, CDCl_3_) *δ* 164.9 (C=O), 164.1 (Ar-C), 162.2 (Ar-C), 151.1 (Ar-C), 145.1 (Ar-C), 139.3 (CH), 138.0 (Ar-C), 137.2 (CH), 130.7 (Ar-C), 124.4 (Ar-C), 122.7 (Ar-C), 120.9 (Ar-C), 118.7 (Ar-C), 117.6 (CH), 116.3 (CH), 114.7 (Ar-C), 112.9 (Ar-C), 56.0 (OCH_3_), 40.2 (CH_2_); HRMS (ESI-MS) m/z: [M + H]^+^ calcd for C_19_H_17_FO_3_^+^: 313.1243; found: 313.1231; IR (cm^−1^): 2937 (OCH_3_), 1704 (C=O), 1165 (Ar-C=C), 1325 (F).

***4-allyl-2-methoxyphenyl (E)-3-(4-fluorophenyl)acrylate* (c10).** Yellow soild; yield 50% (the crude was purified using a mixture of PE/EtOAc 100:1); mp: 270.4–270.6 °C; ^1^H NMR (500 MHz, CDCl_3_) *δ* 7.83 (d, *J* = 16.0 Hz, 1H, Ar-H), 7.58 (d, J = 5.4 Hz, 1H, Ar-H), 7.57 (d, *J* = 5.6 Hz, 1H, CH), 7.12 (s, 1H, Ar-H), 7.10 (d, *J* = 8.6 Hz, 1H, Ar-H), 7.03 (d, *J* = 8.0 Hz, 1H, Ar-H), 6.82 (s, 1H, Ar-H), 6.81 (d, *J* = 8.1 Hz, 1H, Ar-H), 6.60 (d, *J* = 16.0 Hz, 1H, CH), 5.98 (td, *J* = 16.8, 6.7 Hz, 1H, CH), 5.16–5.09 (m, 2H, CH_2_), 3.83 (s, 3H, OCH_3_), 3.40 (d, *J* = 6.7 Hz, 2H, CH_2_); ^13^C NMR (126 MHz, CDCl_3_) *δ* 165.1 (C=O), 163.2 (Ar-C), 151.1 (Ar-C), 145.2 (CH), 139.2 (Ar-C), 138.0 (Ar-C), 137.2 (CH), 130.7 (Ar-C), 130.4 (Ar-C), 130.3 (Ar-C), 122.7 (Ar-C), 120.8 (Ar-C), 117.0 (CH), 116.4 (CH), 116.3 (Ar-C), 116.2 (Ar-C), 112.9 (Ar-C), 56.0 (OCH_3_), 40.3 (CH_2_). HRMS (ESI-MS) m/z: [M + H]^+^ calcd for C_19_H_17_FO_3_^+^: 313.1243; found: 313.1231; IR (cm^−1^): 2937 (OCH_3_), 1704 (C=O), 1165 (Ar-C=C), 1323 (F).

***4-allyl-2-methoxyphenyl (E)-3-(2-bromophenyl)acrylate* (c11)**. Yellow soild; yield 54% (the crude was purified using a mixture of PE/EtOAc 100:1); mp: 296.3–296.5 °C; ^1^H NMR (500 MHz, CDCl_3_) *δ* 8.25 (d, *J* = 15.9 Hz, 1H, Ar-H), 7.70 (d, *J* = 7.9 Hz, 1H, Ar-H), 7.64 (d, *J* = 8.1 Hz, 1H, CH), 7.36 (t, *J* = 7.6 Hz, 1H, Ar-H), 7.26 (d, *J* = 8.0 Hz, 1H, Ar-H), 7.05 (d, *J* = 8.0 Hz, 1H, Ar-H), 6.83 (s, 1H, Ar-H), 6.81 (d, *J* = 7.9 Hz, 1H, Ar-H), 6.63 (d, *J* = 16.0 Hz, 1H, CH), 6.04 (td, *J* = 16.8, 6.7 Hz, 1H, CH), 5.16–5.09 (m, 2H, CH2), 3.84 (s, 3H, OCH3), 3.41 (d, *J* = 6.8 Hz, 2H, CH2); ^13^C NMR (126 MHz, CDCl_3_) *δ* 164.7 (C=O), 151.1 (Ar-C), 144.8 (CH), 139.2 (Ar-C), 138.0 (CH), 137.2 (Ar-C), 134.5 (Ar-C), 133.7 (Ar-C), 131.6 (Ar-C), 128.0 (Ar-C), 127.9 (Ar-C), 125.7 (Ar-C), 122.7 (Ar-C), 120.8 (Ar-C), 120.0 (CH), 116.1 (CH), 112.9 (Ar-C), 56.0 (OCH_3_), 40.2 (CH_2_); HRMS (ESI-MS) m/z: [M + H]^+^ calcd for C_19_H_17_BrO_3_^+^: 373.0442; found: 373.0430 IR (cm^−1^): 2937 (OCH_3_), 1704 (C=O), 1165 (Ar-C=C), 568 (Br).

***4-allyl-2-methoxyphenyl (E)-3-(3-bromophenyl)acrylate* (c12)**. White soild; yield 61% (the crude was purified using a mixture of PE/EtOAc 100:1); mp: 272.6–272.8 °C; ^1^H NMR (500 MHz, CDCl_3_) *δ* 7.78 (d, *J* = 16.0 Hz, 1H, Ar-H), 7.73 (s, 1H, Ar-H), 7.54 (d, *J* = 7.9 Hz, 1H, CH), 7.50 (d, *J* = 7.8 Hz, 1H, Ar-H), 7.29 (t, *J* = 7.9 Hz, 1H, Ar-H), 7.02 (d, *J* = 7.9 Hz, 1H, Ar-H), 6.82 (s, 1H, Ar-H), 6.80 (d, *J* = 8.0 Hz, 1H, Ar-H), 6.66 (d, *J* = 16.0 Hz, 1H, CH), 5.98 (td, *J* = 16.8, 6.8 Hz, 1H, CH), 5.16–5.09 (m, 2H, CH_2_), 3.83 (s, 3H, OCH_3_), 3.40 (d, *J* = 6.7 Hz, 2H, CH2); ^13^C NMR (126 MHz, CDCl_3_) *δ* 164.8 (C=O), 151.0 (Ar-C), 144.8 (CH), 139.2 (Ar-C), 138.0 (Ar-C), 137.2 (CH_2_), 136.5 (Ar-C), 133.5 (Ar-C), 131.1 (Ar-C), 130.6 (Ar-C), 127.0 (Ar-C), 123.2 (Ar-C), 122.7 (Ar-C), 120.9 (CH), 118.8 (CH), 116.3 (Ar-C), 112.9 (Ar-C), 56.0 (OCH_3_), 40.3 (CH); HRMS (ESI-MS) m/z: [M + H]^+^ calcd for C_19_H_17_BrO_3_^+^: 373.0442; found: 313.0433; IR (cm^−1^): 2937 (OCH_3_), 1704 (C=O), 1165 (Ar-C=C), 559 (Br).

***4-allyl-2-methoxyphenyl (E)-3-(4-bromophenyl)acrylate* (c13)**. White soild; yield 58% (the crude was purified using a mixture of PE/EtOAc 100:1); mp: 239.9–340.9 °C; ^1^H NMR (500 MHz, CDCl_3_) *δ* 7.80 (d, J = 16.1 Hz, 1H, Ar-H), 7.56 (s, 1H, Ar-H), 7.54 (s, 1H, CH), 7.45 (s, 1H, Ar-H), 7.43 (s, 1H, Ar-H), 7.02 (d, *J* = 7.9 Hz, 1H, Ar-H), 6.82 (s, 1H, Ar-H), 6.80 (d, *J* = 8.0 Hz, 1H, Ar-H), 6.66 (d, *J* = 16.0 Hz, 1H, CH), 5.98 (td, *J* = 16.9, 6.7 Hz, 1H, CH), 5.15–5.09 (m, 2H, CH_2_), 3.83 (s, 3H, OCH_3_), 3.40 (d, *J* = 6.7 Hz, 2H, CH_2_); ^13^C NMR (126 MHz, CDCl_3_) *δ* 165.3 (C=O), 151.3 (Ar-C), 145.4 (CH), 139.5 (Ar-C), 138.2 (Ar-C), 137.4 (CH), 133.6 (Ar-C), 132.6 (Ar-2C), 130.0 (Ar-2C), 125.3 (Ar-C), 123.0 (Ar-C), 121.1 (CH), 118.2 (CH), 116.6 (Ar-C), 113.1 (Ar-C), 56.3 (OCH_3_), 40.5 (CH_2_); HRMS (ESI-MS) m/z: [M + H]^+^ calcd for C_19_H_17_BrO_3_^+^: 373.0442; found: 373.0429; IR (cm^−1^): 2937 (OCH_3_), 1704 (C=O), 1165 (Ar-C=C), 571 (Br).

***4-allyl-2-methoxyphenyl (E)-3-(3-hydroxyphenyl)acrylate* (c14)**. White soild; yield 51% (the crude was purified using a mixture of PE/EtOAc 40:1); mp: 322.2–322.4 °C; ^1^H NMR (500 MHz, CDCl_3_) *δ* 7.80 (d, *J* = 15.9 Hz, 1H, Ar-H), 7.25 (t, *J* = 7.9 Hz, 1H, Ar-H), 7.12 (d, *J* = 7.9 Hz, 1H, CH), 7.03 (d, *J* = 7.9 Hz, 1H, Ar-H), 7.00 (t, *J* = 2.1 Hz, 1H, Ar-H), 6.86 (d, *J* = 8.1 Hz, 1H, Ar-H), 6.82 (s, 1H, Ar-H), 6.81–6.78 (m, 1H, Ar-H), 6.61 (d, *J* = 15.9 Hz, 1H, CH), 6.01–5.92 (m, 1H, CH), 5.14–5.08 (m, 2H, CH_2_), 3.82 (s, 3H, OCH_3_), 3.38 (d, *J* = 6.7 Hz, 2H, CH2); ^13^C NMR (126 MHz, CDCl_3_) *δ* 165.7 (C=O), 156.3 (Ar-C), 151.0 (Ar-C), 146.7 (Ar-C), 139.3 (CH), 138.0 (Ar-C), 137.2 (CH), 135.8 (Ar-C), 130.2 (Ar-C), 122.7 (Ar-C), 121.0 (Ar-C), 120.9 (Ar-C), 118.0 (Ar-C), 117.3 (CH), 116.3 (CH), 114.8 (Ar-C), 112.9 (Ar-C), 56.0 (OCH_3_), 40.2 (CH_2_); HRMS (ESI-MS) m/z: [M + H]^+^ calcd for C_19_H_18_O_4_^+^: 311.1286; found: 311.1275; IR (cm^−1^): 3376 (OH), 2937 (OCH_3_), 1704 (C=O), 1165 (Ar-C=C).

***4-allyl-2-methoxyphenyl (E)-3-(4-hydroxyphenyl)acrylate* (c15)**. White soild; yield 47% (the crude was purified using a mixture of PE/EtOAc 40:1); mp: 296.3–296.5 °C; ^1^H NMR (500 MHz, CDCl3) δ 7.81 (d, *J* = 15.9 Hz, 1H, Ar-H), 7.46 (d, 1H, Ar-H), 7.45 (d, 1H, CH), 7.03 (d, *J* = 7.9 Hz, 1H, Ar-H), 6.84 (d, 1H, Ar-H), 6.82–6.81 (m, 1H, Ar-H), 6.79 (s, 1H, Ar-H), 6.51 (d, *J* = 15.9 Hz, 1H, Ar-H), 5.97 (td, *J* = 16.8, 6.7 Hz, 1H, CH), 5.83 (s, 1H, CH), 5.15–5.07 (m, 2H, CH_2_), 3.82 (s, 3H, OCH_3_), 3.39 (d, *J* = 6.7 Hz, 2H, CH_2_); ^13^C NMR (126 MHz, CDCl_3_) *δ* 166.0 (C=O), 158.3 (Ar-C), 151.1 (Ar-C), 146.6 (CH), 139.2 (Ar-C), 138.0 (CH), 137.2 (Ar-C), 130.4 (Ar-2C), 127.0 (Ar-C), 122.8 (Ar-C), 120.9 (Ar-C), 116.3 (CH), 116.1 (Ar-2C), 114.3 (CH), 112.9 (Ar-C), 56.0 (OCH_3_), 40.3 (CH_2_); HRMS (ESI-MS) m/z: [M + H]^+^ calcd for C_19_H_18_O_4_^+^: 311.1286; found: 311.1273; IR (cm^−1^): 3371 (OH), 2937 (OCH_3_), 1704 (C=O), 1165 (Ar-C=C).

***4-allyl-2-methoxyphenyl (E)-3-(2-nitrophenyl)acrylate* (c16)**. Yellow soild; yield 42% (the crude was purified using a mixture of PE/EtOAc 100:1); mp: 295.2–295.4 °C; ^1^H NMR (500 MHz, CDCl_3_) *δ* 8.31 (d, *J* = 15.8 Hz, 1H, Ar-H), 8.07 (d, *J* = 8.1 Hz, 1H, Ar-H), 7.73 (d, *J* = 6.2 Hz, 1H, CH), 7.69 (t, *J* = 7.6 Hz, 1H, Ar-H), 7.58 (t, *J* = 7.8 Hz, 1H, Ar-H), 7.04 (d, *J* = 7.9 Hz, 1H, Ar-H), 6.84–6.82 (m, 1H, Ar-H), 6.81 (d, *J* = 8.0 Hz, 1H, Ar-H), 6.59 (d, *J* = 15.8 Hz, 1H, CH), 5.98 (td, *J* = 16.9, 6.7 Hz, 1H, CH), 5.14–5.09 (m, 2H, CH_2_), 3.84 (s, 3H, OCH_3_), 3.40 (d, *J* = 6.5 Hz, 2H, CH_2_); ^13^C NMR (126 MHz, CDCl_3_) *δ* 164.1 (C=O), 151.0 (Ar-C), 148.5 (Ar-C), 141.7 (CH), 139.3 (Ar-C), 138.0 (Ar-C), 137.2 (CH), 133.8 (Ar-C), 130.7 (Ar-C), 130.6 (Ar-C), 129.4 (Ar-C), 125.1 (Ar-C), 122.7 (Ar-C), 122.4 (Ar-C), 120.8 (CH), 116.3 (CH), 112.9 (Ar-C), 56.0 (OCH_3_), 40.3 (CH_2_); HRMS (ESI-MS) m/z: [M + H]^+^ calcd for C_19_H_17_NO_5_^+^: 340.1181; found: 340.1176; IR (cm^−1^): 2937 (OCH_3_), 1704 (C=O), 1424 (NO_2_), 1165 (Ar-C=C).

***4-allyl-2-methoxyphenyl (E)-3-(3-nitrophenyl)acrylate* (c17)**. White soild; yield 51% (the crude was purified using a mixture of PE/EtOAc 100:1); mp: 255.5–256.1 °C; ^1^H NMR (500 MHz, CDCl_3_) *δ* 8.44 (t, *J* = 1.9 Hz, 1H, Ar-H), 8.26 (d, *J* = 9.9 Hz, 1H, Ar-H), 7.90 (d, *J* = 11.4 Hz, 1H, CH), 7.88 (d, *J* = 3.2 Hz, 1H, Ar-H), 7.61 (t, *J* = 8.0 Hz, 1H, Ar-H), 7.03 (d, *J* = 8.0 Hz, 1H, Ar-H), 6.83 (d, *J* = 1.9 Hz, 1H, Ar-H), 6.81 (d, *J* = 2.8 Hz, 1H, Ar-H), 6.79 (d, *J* = 11.1 Hz, 1H, CH), 5.98 (td, *J* = 16.9, 6.7 Hz, 1H, CH), 5.16–5.08 (m, 2H, CH_2_), 3.83 (s, 3H, OCH_3_), 3.40 (d, *J* = 6.7 Hz, 2H, CH_2_); ^13^C NMR (126 MHz, CDCl_3_) *δ* 164.4 (C=O), 151.7 (Ar-C), 148.8 (Ar-C), 143.5 (CH), 139.4 (Ar-C), 137.9 (Ar-C), 137.1 (CH), 136.1 (Ar-C), 133.9 (Ar-C), 130.2 (Ar-C), 124.9 (Ar-C), 122.8 (Ar-C), 122.6 (Ar-C), 120.8 (Ar-C), 120.5 (CH), 116.4 (CH), 112.9 (Ar-C), 56.0 (OCH_3_), 40.3 (CH_2_); HRMS (ESI-MS) m/z: [M + H]^+^ calcd for C_19_H_17_NO_5_^+^: 340.1181; found: 340.1178; IR (cm^−1^): 2937 (OCH_3_), 1704 (C=O), 1421 (NO_2_), 1165 (Ar-C=C).

***4-allyl-2-methoxyphenyl (E)-3-(4-nitrophenyl)acrylate* (c18)**. Yellow soild; yield 58% (the crude was purified using a mixture of PE/EtOAc 80:1); mp: 291.2–292.3 °C; ^1^H NMR (500 MHz, CDCl_3_) *δ* 8.28 (d, *J* = 1.8 Hz, 1H, Ar-H), 8.27 (d, *J* = 2.0 Hz, 1H, Ar-H), 7.89 (d, *J* = 16.0 Hz, 1H, CH), 7.74 (d, *J* = 2.0 Hz, 1H, Ar-H), 7.72 (d, *J* = 2.1 Hz, 1H, Ar-H), 7.03 (d, *J* = 8.0 Hz, 1H, Ar-H), 6.83 (d, *J* = 1.3 Hz, 1H, Ar-H), 6.81 (d, *J* = 5.7 Hz, 1H, Ar-H), 6.79 (d, *J* = 13.7 Hz, 1H, CH), 5.97 (td, *J* = 16.8, 6.7 Hz, 1H, CH), 5.16–5.09 (m, 2H, CH_2_), 3.83 (s, 3H, OCH_3_), 3.40 (d, *J* = 6.7 Hz, 2H, CH_2_); ^13^C NMR (126 MHz, CDCl_3_) *δ* 164.3 (C=O), 151.0 (Ar-C), 148.8 (CH), 143.5 (Ar-C), 140.5 (CH), 139.5 (Ar-C), 137.8 (Ar-C), 137.0 (Ar-C), 129.0 (Ar-2C), 124.4 (Ar-2C), 122.6 (Ar-C), 121.6 (Ar-C), 120.9 (CH), 116.4 (CH), 112.8 (Ar-C), 56.0 (OCH_3_), 40.3 (CH_2_); HRMS (ESI-MS) m/z: [M + H]^+^ calcd for C_19_H_17_NO_5_^+^: 340.1181; found: 340.1178; IR (cm^−1^): 2937 (OCH_3_), 1704 (C=O), 1424 (NO_2_), 1165 (Ar-C=C).

***4-allyl-2-methoxyphenyl (E)-3-(2-methoxyphenyl)acrylate* (c19)**. White soild; yield 49% (the crude was purified using a mixture of PE/EtOAc 80:1); mp: 250.3–250.8 °C; ^1^H NMR (500 MHz, CDCl_3_) *δ* 8.17 (d, *J* = 16.1 Hz, 1H, Ar-H), 7.57 (d, *J* = 7.7 Hz, 1H, Ar-H), 7.38 (t, *J* = 8.7 Hz, 1H, CH), 7.03 (d, *J* = 7.9 Hz, 1H, Ar-H), 6.99 (t, *J* = 7.5 Hz, 1H, Ar-H), 6.94 (d, *J* = 8.3 Hz, 1H, Ar-H), 6.82 (d, *J* = 1.9 Hz, 1H, Ar-H), 6.81 (s, 1H, Ar-H), 6.78 (d, *J* = 9.2 Hz, 1H, CH), 5.99 (td, *J* = 16.8, 6.7 Hz, 1H, CH), 5.16–5.08 (m, 2H, CH_2_), 3.90 (s, 3H, CH_3_), 3.83 (s, 3H, OCH_3_), 3.40 (d, *J* = 6.7 Hz, 2H, CH_2_); ^13^C NMR (126 MHz, CDCl_3_) *δ* 165.8 (C=O), 158.7 (Ar-C), 151.2 (Ar-C), 142.1 (CH), 139.0 (Ar-C), 138.2 (CH), 137.3 (Ar-C), 131.9 (Ar-C), 129.5 (Ar-C), 123.4 (Ar-C), 122.8 (Ar-C), 120.9 (Ar-C), 120.8 (Ar-C), 117.7 (CH), 116.3 (CH), 112.9 (Ar-C), 111.3 (Ar-C), 56.0 (OCH_3_), 55.6 (OCH_3_), 40.3 (CH_2_); HRMS (ESI-MS) m/z: [M + H]^+^ calcd for C_20_H_20_O_4_^+^: 325.1437; found: 325.1432; IR (cm^−1^): 2937, 2873 (OCH_3_), 1704 (C=O), 1165 (Ar-C=C).

***4-allyl-2-methoxyphenyl (E)-3-(3-methoxyphenyl)acrylate* (c20)**. White soild; yield 60% (the crude was purified using a mixture of PE/EtOAc 100:1); mp: 246.3–246.4 °C; ^1^H NMR (500 MHz, CDCl_3_) *δ* 7.84 (d, *J* = 16.0 Hz, 1H, Ar-H), 7.33 (t, *J* = 7.9 Hz, 1H, Ar-H), 7.18 (d, *J* = 7.8 Hz, 1H, CH), 7.11 (t, *J* = 2.1 Hz, 1H, Ar-H), 7.03 (d, *J* = 8.0 Hz, 1H, Ar-H), 6.97 (d, *J* = 8.6 Hz, 1H, Ar-H), 6.83 (d, *J* = 1.9 Hz, 1H, Ar-H), 6.81 (d, *J* = 8.0 Hz, 1H, Ar-H), 6.67 (d, *J* = 15.9 Hz, 1H, CH), 5.98 (td, *J* = 16.9, 6.8 Hz, 1H, CH), 5.16–5.09 (m, 2H, CH_2_), 3.85 (s, 3H, OCH_3_), 3.83 (s, 3H, CH_3_), 3.40 (d, *J* = 6.7 Hz, 2H, CH_2_); ^13^C NMR (126 MHz, CDCl_3_) *δ* 165.2 (C=O), 160.0 (Ar-C), 151.1 (Ar-C), 146.5 (CH), 139.1 (Ar-C), 138.1 (CH), 137.2 (Ar-C), 135.8 (Ar-C), 130.1 (Ar-C), 122.8 (Ar-C), 121.2 (Ar-C), 120.9 (Ar-C), 117.5 (Ar-C), 116.7 (CH), 116.3 (CH), 113.1 (Ar-C), 112.9 (Ar-C), 56.0 (OCH_3_), 55.4 (OCH_3_), 40.2 (CH_2_); HRMS (ESI-MS) m/z: [M + H]^+^ calcd for C_20_H_20_O_4_^+^: 325.1437; found: 325.1432; IR (cm^−1^): 2937, 2867 (OCH_3_), 1704 (CO), 1165 (PH-C=C).

***4-allyl-2-methoxyphenyl (E)-3-(4-methoxyphenyl)acrylate* (c21)**. White soild; yield 62% (the crude was purified using a mixture of PE/EtOAc 100:1); mp: 328.6–329.0 °C; ^1^H NMR (500 MHz, CDCl_3_) *δ* 7.83 (d, *J* = 15.9 Hz, 1H, Ar-H), 7.55 (s, 1H, Ar-H), 7.53 (d, 1H, CH), 7.03 (d, *J* = 7.9 Hz, 1H, Ar-H), 6.94 (d, *J* = 2.1 Hz, 1H, Ar-H), 6.93 (d, *J* = 2.0 Hz, 1H, Ar-H), 6.82 (d, *J* = 1.9 Hz, 1H, Ar-H), 6.80 (d, *J* = 7.9 Hz, 1H, Ar-H), 6.54 (d, *J* = 15.9 Hz, 1H, CH), 5.98 (td, *J* = 16.9, 6.7 Hz, 1H, CH), 5.17–5.08 (m, 2H, CH_2_), 3.85 (s, 3H, CH_3_), 3.83 (s, 3H, OCH_3_), 3.40 (d, *J* = 6.8 Hz, 2H, CH_2_); ^13^C NMR (126 MHz, CDCl_3_) *δ* 165.6 (C=O), 161.7 (Ar-C), 151.2 (Ar-C), 146.3 (CH), 139.0 (Ar-C), 138.2 (CH), 137.2 (Ar-C), 130.1 (Ar-2C), 127.2 (Ar-C), 122.9 (Ar-C), 120.9 (Ar-C), 116.3 (CH), 114.6 (CH), 114.5 (Ar-2C), 112.8 (Ar-C), 56.0 (OCH_3_), 55.5 (OCH_3_), 40.2 (CH_2_); HRMS (ESI-MS) m/z: [M + H]^+^ calcd for C_20_H_20_O_4_^+^: 325.1437; found: 325.1432; IR (cm^−1^): 2937, 2871 (OCH_3_), 1704 (C=O), 1165 (Ar-C=C).

***4-allyl-2-methoxyphenyl (E)-3-(2-(trifluoromethyl)phenyl)acrylate* (c22)**. White soild; yield 47% (the crude was purified using a mixture of PE/EtOAc 100:1); mp: 339.4–340.1 °C; ^1^H NMR (500 MHz, CDCl_3_) *δ* 8.25 (d, *J* = 15.8 Hz, 1H, Ar-H, 7.80 (d, *J* = 7.8 Hz, 1H, Ar-H), 7.73 (d, *J* = 7.9 Hz, 1H, CH), 7.61 (t, *J* = 7.7 Hz, 1H, Ar-H), 7.54–7.49 (m, 1H, Ar-H), 7.05 (d, *J* = 7.9 Hz, 1H, Ar-H), 6.83 (d, *J* = 1.9 Hz, 1H, Ar-H), 6.81 (d, *J* = 8.0 Hz, 1H, Ar-H), 6.65 (d, *J* = 15.8 Hz, 1H, CH), 5.98 (td, *J* = 16.8, 6.7 Hz, 1H, CH), 5.16–5.09 (m, 2H, CH_2_), 3.84 (s, 3H, OCH_3_), 3.41 (d, J = 6.8 Hz, 2H, CH_2_); ^13^C NMR (126 MHz, CDCl_3_) *δ* 164.4 (C=O), 151.1 (Ar-C), 141.9 (Ar-C), 139.2 (CH), 138.0 (Ar-C), 137.2 (CH), 133.3 (Ar-C), 132.3 (Ar-C), 130.0 (Ar-C), 129.3 (Ar-C), 128.2 (Ar-C), 126.4 (Ar-C), 125.1 (Ar-C), 122.7 (Ar-C), 121.6 (CF_3_), 120.9 (CH), 116.3 (CH), 113.9 (Ar-C), 56.0 (OCH_3_), 40.3 (CH_2_); HRMS (ESI-MS) m/z: [M + H]^+^ calcd for C_20_H_17_F_3_O_3_^+^: 363.1205; found: 363.1198; IR (cm^−1^): 2937 (OCH_3_), 1704 (C=O), 1332 (CF_3_), 1165 (Ar-C=C).

***4-allyl-2-methoxyphenyl (E)-3-(3-(trifluoromethyl)phenyl)acrylate* (c23)**. Yellow soild; yield 69% (the crude was purified using a mixture of PE/EtOAc 100:1); mp: 339.1–339.3 °C; ^1^H NMR (500 MHz, CDCl_3_) *δ* 7.88 (d, *J* = 16.0 Hz, 1H, Ar-H), 7.83 (s, 1H, Ar-H), 7.76 (d, *J* = 7.8 Hz, 1H, CH), 7.67 (d, *J* = 7.1 Hz, 1H, Ar-H), 7.55 (t, *J* = 7.8 Hz, 1H, Ar-H), 7.03 (d, *J* = 8.0 Hz, 1H, Ar-H), 6.83 (d, *J* = 2.0 Hz, 1H, Ar-H), 6.81 (dd, *J* = 8.0, 1.9 Hz, 1H, Ar-H), 6.74 (d, *J* = 16.0 Hz, 1H, CH), 5.98 (td, *J* = 16.9, 6.7 Hz, 1H, CH), 5.16–5.09 (m, 2H, CH_2_), 3.83 (s, 3H, OCH_3_), 3.40 (d, *J* = 6.7 Hz, 2H, CH_2_); ^13^C NMR (126 MHz, CDCl_3_) *δ* 164.7 (C=O), 151.0 (Ar-C), 144.7 (CH), 139.3 (Ar-C), 138.0 (CH), 137.2 (Ar-C), 135.2 (Ar-C), 131.8 (Ar-C), 131.4 (Ar-C), 129.7 (Ar-C), 127.1 (CF_3_), 125.0 (Ar-C), 122.7 (Ar-C), 120.9 (Ar-C), 119.3 (Ar-C), 116.4 (CH), 112.9 (CH), 56.0 (Ar-C), 55.9 (OCH_3_), 40.3 (CH_2_); HRMS (ESI-MS) m/z: [M + H]^+^ calcd for C_20_H_17_F_3_O_3_^+^: 363.1205; found: 363.1199; IR (cm^−1^): 2937 (OCH_3_), 1704 (C=O), 1330 (CF_3_), 1165 (Ar-C=C).

***4-allyl-2-methoxyphenyl (E)-3-(4-(trifluoromethyl)phenyl)acrylate* (c24)**. White soild; yield 42% (the crude was purified using a mixture of PE/EtOAc 60:1); mp: 343.2–343.3 °C; ^1^H NMR (500 MHz, CDCl_3_) *δ* 7.88 (d, *J* = 16.0 Hz, 1H, Ar-H), 7.68 (s, 1H, Ar-H), 7.68 (s, 1H, CH), 7.68 (s, 1H, Ar-H), 7.68 (s, 1H, Ar-H), 7.03 (d, *J* = 7.9 Hz, 1H, Ar-H), 6.83 (d, *J* = 1.9 Hz, 1H, Ar-H), 6.81 (dd, *J* = 8.0, 1.9 Hz, 1H, Ar-H), 6.75 (d, *J* = 16.0 Hz, 1H, CH), 5.98 (td, *J* = 16.8, 6.7 Hz, 1H, CH), 5.16–5.06 (m, 2H, CH_2_), 3.83 (s, 3H, OCH_3_), 3.41 (d, *J* = 6.7 Hz, 2H, CH_2_); ^13^C NMR (126 MHz, CDCl_3_) *δ* 164.7 (C=O), 151.0 (Ar-C), 144.6 (CH), 139.4 (Ar-C), 137.0 (Ar-C), 137.8 (CH), 137.1 (Ar-C), 132.0 (Ar-C), 128.5 (Ar-2C), 126.1 (Ar-2C), 126.0 (CF_3_), 122.7 (Ar-C), 120.9 (Ar-C), 119.9 (CH), 116.4 (CH), 112.9 (Ar-C), 56.0 (OCH_3_), 40.3 (CH_2_); HRMS (ESI-MS) m/z: [M + H]^+^ calcd for C_20_H_17_F_3_O_3_^+^: 363.1205; found: 363.1200; IR (cm-1): 2937 (OCH_3_), 1704 (C=O), 1331 (CF_3_), 1165 (Ar-C=C).

***4-allyl-2-methoxyphenyl (E)-3-(4-(dimethylamino)phenyl)acrylate* (c25)**. Yellow soild; yield 29% (the crude was purified using a mixture of PE/EtOAc 40:1); mp: 328.6–329.1 °C; ^1^H NMR (500 MHz, CDCl_3_) *δ* 7.80 (d, *J* = 15.8 Hz, 1H, Ar-H), 7.49 (s, 1H, Ar-H), 7.47 (s, 1H, CH), 7.02 (d, *J* = 7.9 Hz, 1H, Ar-H), 6.81 (d, *J* = 1.9 Hz, 1H, Ar-H), 6.79 (dd, *J* = 7.9, 1.9 Hz, 1H, Ar-H), 6.70 (s, 1H, Ar-H), 6.68 (d, 1H, Ar-H), 6.44 (d, *J* = 15.8 Hz, 1H, CH), 5.98 (td, *J* = 16.8, 6.7 Hz, 1H, CH), 5.17–5.07 (m, 2H, CH_2_), 3.83 (s, 3H, OCH_3_), 3.39 (d, *J* = 6.8 Hz, 2H, CH_2_), 3.04 (s, 6H, CH_3_, CH_3_); ^13^C NMR (126 MHz, CDCl_3_) *δ* 166.2 (C=O), 152.0 (CH), 151.3 (Ar-C), 147.2 (Ar-C), 138.8 (CH), 138.4 (Ar-C), 137.3 (Ar-C), 130.2 (Ar-C), 123.0 (Ar-C), 120.8 (Ar-C), 116.2 (CH), 112.8 (CH), 112.0 (Ar-C), 111.3 (Ar-C), 56.0 (OCH_3_), 40.3 ((CH_3_)_2_), 40.3 (CH_2_); HRMS (ESI-MS) m/z: [M + H]^+^ calcd for C_21_H_23_NO_3_^+^: 338.1754; found: 338.1746; IR (cm^−1^): 2937 (OCH_3_), 2925 (CH_2_), 2968 (CH_3_), 1704 (C=O), 1609 (N), 1165 (Ar-C=C).

***4-allyl-2-methoxyphenyl (E)-3-(4-hydroxy-3-methoxyphenyl)acrylate* (c26)**. White soild; yield 56% (the crude was purified using a mixture of PE/EtOAc 100:1); mp: 274.3–274.8 °C; ^1^H NMR (500 MHz, CDCl_3_) *δ* 7.80 (d, *J* = 15.9 Hz, 1H, Ar-H), 7.13 (d, *J* = 8.1 Hz, 1H, Ar-H), 7.10 (d, *J* = 1.9 Hz, 1H, CH), 7.02 (d, *J* = 7.9 Hz, 1H, Ar-H), 6.94 (d, *J* = 8.2 Hz, 1H, Ar-H), 6.82 (d, *J* = 1.9 Hz, 1H, Ar-H), 6.80 (d, *J* = 8.0 Hz, 1H, Ar-H), 6.52 (d, *J* = 15.9 Hz, 1H, CH), 5.99 (dd, *J* = 16.9, 6.8 Hz, 1H, CH), 5.95–5.87 (m, 1H, CH), 5.16–5.08 (m, 2H, CH_2_), 3.93 (s, 3H, CH_3_), 3.83 (s, 3H, OCH_3_), 3.40 (d, *J* = 6.8 Hz, 2H, CH_2_); ^13^C NMR (126 MHz, CDCl_3_) *δ* 165.6 (C=O), 151.2 (Ar-C), 148.4 (Ar-C), 146.9 (Ar-C), 146.7 (CH), 139.0 (Ar-C), 138.2 (CH), 137.2 (Ar-C), 127.0 (Ar-C), 123.6 (Ar-C), 122.8 (Ar-C), 120.8 (Ar-C), 116.3 (Ar-C), 114.9 (CH), 114.5 (CH), 112.9 (Ar-C), 109.5 (Ar-C), 56.1 (OCH_3_), 56.00 (OCH_3_), 40.3 (CH_2_); HRMS (ESI-MS) m/z: [M + H]^+^ calcd for C_20_H_20_O_5_^+^: 341.1386; found: 341.1380; IR (cm^−1^): 3239 (OH), 2937, 2854 (OCH_3_), 1704 (C=O), 1165 (Ar-C=C).

***4-allyl-2-methoxyphenyl (E)-3-(3,4-dihydroxyphenyl)acrylate* (c27)**. Yellow soild; yield 37% (the crude was purified using a mixture of PE/EtOAc 100:1); mp: 265.5–266.5 °C; ^1^H NMR (500 MHz, CDCl_3_) *δ* 7.72 (d, *J* = 15.8 Hz, 1H, Ar-H), 7.04 (s, 1H, Ar-H), 7.00 (d, *J* = 8.1 Hz, 1H, CH), 6.98 (d, *J* = 7.4 Hz, 1H, Ar-H), 6.82 (s, 1H, Ar-H), 6.79 (d, 1H, Ar-H), 6.77 (d, *J* = 2.0 Hz, 1H, Ar-H), 6.42 (d, *J* = 15.9 Hz, 1H, CH), 5.98–5.91 (m, 1H, CH), 5.14–5.07 (m, 2H, CH_2_), 3.79 (s, 3H, OCH_3_), 3.37 (d, *J* = 6.7 Hz, 2H, CH_2_); ^13^C NMR (126 MHz, CDCl_3_) *δ* 166.1 (C=O), 150.8 (Ar-C), 146.9 (CH), 146.8 (Ar-C), 143.9 (Ar-C), 139.0 (Ar-C), 137.8 (CH), 136.9 (Ar-C), 127.0 (Ar-C), 122.7 (Ar-C), 122.5 (Ar-C), 120.7 (Ar-C), 116.1 (Ar-C), 115.3 (CH), 114.4 (CH), 114.0 (Ar-C), 112.7 (Ar-C), 55.8 (OCH_3_), 40.0 (CH_2_); HRMS (ESI-MS) m/z: [M + H]^+^ calcd for C_19_H_18_O_5_^+^: 327.1236; found: 327.1225; IR (cm^−1^): 3217, 3186 (OH), 2937 (OCH_3_), 1704 (C=O), 1165 (Ar-C=C).

***4-allyl-2-methoxyphenyl (E)-3-(4-chloro-3-fluorophenyl)acrylate* (c28)**. Yellow soild; yield 40% (the crude was purified using a mixture of PE/EtOAc 80:1); mp: 264.2–264.9 °C; ^1^H NMR (500 MHz, CDCl_3_) δ 7.76 (d, *J* = 16.0 Hz, 1H, Ar-H), 7.63 (dd, *J* = 7.0, 2.2 Hz, 1H, Ar-H), 7.45 (ddd, *J* = 8.6, 4.5, 2.2 Hz, 1H, CH), 7.19 (t, *J* = 8.6 Hz, 1H, Ar-H), 7.02 (d, *J* = 8.0 Hz, 1H, Ar-H), 6.82 (d, *J* = 2.0 Hz, 1H, Ar-H), 6.80 (d *J* = 8.0 Hz, 1H, Ar-H), 6.60 (d, *J* = 16.0 Hz, 1H, CH), 5.98 (td, *J* = 16.8, 6.7 Hz, 1H, CH), 5.16–5.09 (m, 2H, CH_2_), 3.83 (s, 3H, OCH_3_), 3.40 (d, *J* = 6.8 Hz, 2H, CH_2_); ^13^C NMR (126 MHz, CDCl_3_) *δ* 164.7 (C=O), 160.4 (Ar-C), 158.3 (Ar-C), 151.0 (CH), 143.8 (Ar-C), 139.2 (CH), 137.9 (Ar-C), 137.2 (Ar-C), 131.8 (Ar-C), 130.4 (Ar-C), 128.3 (Ar-C), 122.7 (Ar-C), 120.9 (Ar-C), 118.4 (CH), 117.4 (CH), 116.3 (Ar-C), 112.8 (Ar-C), 56.0 (OCH_3_), 40.3 (CH_2_); HRMS (ESI-MS) m/z: [M + H]^+^ calcd for C_19_H_16_ClFO_3_^+^: 347.0853; found: 347.0841; IR (cm^−1^): 2937 (OCH3), 1704 (C=O), 1165 (Ar-C=C), 1046 (F), 728 (Cl).

***4-allyl-2-methoxyphenyl (E)-3-(benzo[d][1,3]dioxol-5-yl)acrylate* (c29)**. White soild; yield 55% (the crude was purified using a mixture of PE/EtOAc 100:1); mp: 267.2–268.1 °C; ^1^H NMR (500 MHz, CDCl_3_) *δ* 7.77 (d, *J* = 15.9 Hz, 1H, CH), 7.09 (d, *J* = 1.7 Hz, 1H, Ar-H), 7.06 (d, *J* = 8.1 Hz, 1H, Ar-H), 7.02 (d, *J* = 8.0 Hz, 1H, Ar-H), 6.84 (d, *J* = 8.0 Hz, 1H, Ar-H), 6.82 (d, *J* = 1.9 Hz, 1H, Ar-H), 6.80 (d, *J* = 7.9 Hz, 1H, Ar-H), 6.49 (d, *J* = 15.9 Hz, 1H, CH), 6.02 (s, 2H, CH_2_), 6.01–5.90 (m, 1H, CH), 5.15–5.06 (m, 2H, CH_2_), 3.83 (s, 3H, OCH_3_), 3.40 (d, *J* = 6.7 Hz, 2H, CH_2_); ^13^C NMR (126 MHz, CDCl_3_) *δ* 165.5 (C=O), 151.2 (Ar-C), 150.0 (Ar-C), 148.5 (Ar-C), 146.3 (Ar-C), 139.1 (CH), 138.1 (Ar-C), 137.2 (CH), 128.9 (Ar-C), 125.0 (Ar-C), 122.8 (Ar-C), 120.8 (Ar-C), 116.3 (CH), 115.1 (CH), 112.9 (Ar-C), 108.7 (Ar-C), 106.8 (Ar-C), 101.8 (CH_2_), 56.0 (OCH_3_), 40.3 (CH_2_); HRMS (ESI-MS) m/z: [M + H]^+^ calcd for C_20_H_18_O_5_^+^: 339.1229 found: 339.1224; IR (cm^−1^): 2937 (OCH3), 1704 (C=O), 1165 (Ar-C=C), 953, 946 (OCH_2_).

#### 3.2.2. Tyrosinase Inhibition and Kinetics Study

Compounds **c1**~**c29** were tested for their tyrosinase inhibitory activities according to previous reports [53,54]. Each compound was dissolved in DMSO and diluted to different concentrations. Tyrosinase and L-DOPA were dissolved in PBS solution (50 mM, pH 6.8). To 140 μL of tyrosinase (final concentration: 66.7 U/mL) solution, 10 μL of compound was added and incubated for 5 min at 25 °C, followed by the addition of 50 µL of L-DOPA (final concentration: 2 mM). Then, absorbance of the mixture was read at 475 nm. The inhibitory activity of each compound was calculated using DMSO as a blank control. Kojic acid was also used as the positive control.

The kinetics studies were carried out using the same experimental method as described above. For enzyme kinetics, the absorbance of the mixture containing different concentrations of tyrosinase (final concentration: 66.7 U/mL) and compound c27 (0, 2, 3, and 4 μM) was recorded at 475 nm, respectively. For substrate kinetics, the absorbance of the mixture containing different concentrations of L-DOPA (final concentration: 2, 4, 6 and 8 mM) and compound **c27** (0, 2, 3 and 4 μM) was recorded at 475 nm, respectively.

#### 3.2.3. Three-Dimensional Fluorescence Spectra Assay

The 3D fluorescence spectra of tyrosinase-compound **c27** mixture was measured according to previous reports [55]. To 3 mL of tyrosinase solution (final concentration: 168 μM) in PBS, 10 µL of compound **c27** (2 mM) was added and incubated for 5 min. Then, the 3D fluorescence spectra analyses were conducted in room temperature. The excitation and emission wavelengths were 200–600 nm and the slit width was 2.5 nm.

#### 3.2.4. Circular Dichroism Spectroscopy Assay

The CD spectra of tyrosinase-compound **c27** mixture were measured according to previous reports [56]. To 190 µL of tyrosinase solution (final concentration: 64.65 μM) in PBS, 10 µL of compound **c27** (1.28, 2.56, and 3.84 mM) was added and incubated for 5 min. Then, the CD spectra were measured at 195~250 nm at room temperature.

#### 3.2.5. Fluorescence Quenching Experiments

An amount of 1 μL of compound **c27** solution was titrated into 2 mL of tyrosinase and incubated for 5 min. Then, the fluorescence spectra were determined at excitation wavelength of 280 nm and temperatures of 303, 307, and 310 K, respectively. The quenching mechanism and parameters were investigated by the Stern–Volmer equation.

#### 3.2.6. Molecular Docking

Molecular docking of tyrosinase–compound **c27** was conducted using SYBYL software according to previous reports [57,58,59,60]. Compound **c27** was treated with hydrogenation and energy minimization with the Gasteiger–Hückel program. The crystal structure of tyrosinase (PDBID: 2Y9X) was obtained from the RCSB Bank. Tyrosinase protein was analyzed with the internal program and we generated the active pocket using ligand mode. Then, the docking program was conducted in the default format to simulate the docking between tyrosinase and compound **c27**. In the docking process, 20 poses were generated for compound **c27**. We selected the pose with the highest score to further analyze the detailed interaction.

## 4. Conclusions

In this study, we synthesized cinnamic acid–eugenol esters **c1**~**c29** and assayed their tyrosinase inhibitory activities. The results showed that all cinnamic acid–eugenol esters **c1**~**c29** exhibited definite tyrosinase inhibitory activities. Compound **c27** was the strongest tyrosinase inhibitor (IC_50_ = 3.07 ± 0.26 μM), and thus validated as a reversible mixed-type inhibitor against tyrosinase. Three-dimensional fluorescence and CD spectra results indicated that compound **c27** could change the conformation and secondary structure of tyrosinase. Fluorescence quenching results showed that compound **c27** quenched tyrosinase fluorescence in a static manner with one binding site. Molecular docking results also revealed the binding interactions between compound **c27** and tyrosinase. Therefore, cinnamic acid–eugenol esters, especially **c27**, could be used as lead compounds to find potential tyrosinase inhibitors.

## Figures and Tables

**Table 1:** Tyrosinase inhibitory activities of cinnamic acid esters **c1**~**c29**.

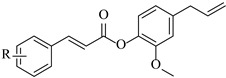			
Compounds	R	Inhibition Rate at 64 μM (%)	IC_50_ (μM)
**c1**	-H	12 ± 1	-
**c2**	-2CH_3_	12 ± 0.3	-
**c3**	-3CH_3_	39 ± 3	-
**c4**	-4CH_3_	15 ± 1	-
**c5**	-2Cl	29 ± 3	-
**c6**	-3Cl	17 ± 2	-
**c7**	-4Cl	24 ± 1	-
**c8**	-2F	17 ± 3	-
**c9**	-3F	17 ± 3	-
**c10**	-4F	17 ± 1	-
**c11**	-2Br	10 ± 0.4	-
**c12**	-3Br	3 ± 0.8	-
**c13**	-4Br	13 ± 1	-
**c14**	-3OH	68 ± 0.7	16.28 ± 1.52
**c15**	-4OH	76 ± 0.2	5.95 ± 1.46
**c16**	-2NO_2_	5 ± 0.1	-
**c17**	-3NO_2_	9 ± 0.8	-
**c18**	-4NO_2_	14 ± 1	-
**c19**	-2CH_3_O	3 ± 0.2	-
**c20**	-3CH_3_O	3 ± 0.1	-
**c21**	-4CH_3_O	7 ± 0.9	-
**c22**	-2CF_3_	6 ± 0.5	-
**c23**	-3CF_3_	5 ± 0.2	-
**c24**	-4CF_3_	2 ± 0.1	-
**c25**	-(CH_3_CH_2_)_2_N	26 ± 1	-
**c26**	-3CH_3_O, -4OH	47 ± 1	-
**c27**	-3,4OH	81 ± 2	3.07 ± 0.28
**c28**	-3F, -4Cl	19 ± 0.2	-
**c29**	-3,4CH_2_O	4 ± 1	-
**Cinnamic acid**			201.4 ± 5.3
**Eugenol**			5521 ± 25
**Kojic acid**		76 ± 2	14.15 ± 0.46

‘‘-” mean not detected.

**Table 2:** The secondary structures contents of tyrosinase.

[Tyrosinase]:[c27]	α-Helix (%)	β-Sheet (%)	β-Turn (%)	Random Coil (%)
1:0	7.10	48.90	17.21	28.40
1:1	7.12	49.00	17.63	28.23
1:2	9.56	49.23	18.37	28.12
1:3	9.85	49.27	18.55	27.94

**Table 3:** Quenching constants of tyrosinase with compound **c27**.

T (K)	*K*_SV_ (×10^4^ L·mol^−1^)	*K*_q_ (×10^12^ L·mol^−1^)	*K*_a_ (×10^4^ L·mol^−1^)	n
304	4.77	4.77	0.05	0.59
307	4.32	4.32	0.93	0.86
310	4.29	4.29	3.85	0.99

**Table 4:** Drug-like properties of compound **c27**.

MF	MW	RB	HBA	HBD	PPSA	Log Po/w	WS
C_19_H_18_O_5_	326.34	7	5	2	75.99	3.10	Poorly

MF (molecular formula), MW (molecular weight, g/mol), RB (rotatable bonds), HBA (H-bond acceptor atoms), HBD (H-bond donor atoms), TPSA (topology polar surface area, Å²), WS (water solubility).

## Data Availability

Not applicable.
